# Retrieval Augmented Therapy Suggestion for Molecular Tumor Boards: Algorithmic Development and Validation Study

**DOI:** 10.2196/64364

**Published:** 2025-03-05

**Authors:** Eliza Berman, Holly Sundberg Malek, Michael Bitzer, Nisar Malek, Carsten Eickhoff

**Affiliations:** 1 Center for Digital Health University Hospital Tuebingen Tuebingen Germany; 2 Center for Personalized Medicine University Hospital Tuebingen Tuebingen Germany; 3 Department of Internal Medicine I University Hospital Tuebingen Tuebingen Germany

**Keywords:** large language models, retrieval augmented generation, LLaMA, precision oncology, molecular tumor board, molecular tumor, LLMs, augmented therapy, MTB, oncology, tumor, clinical trials, patient care, treatment, evidence-based, accessibility to care

## Abstract

**Background:**

Molecular tumor boards (MTBs) require intensive manual investigation to generate optimal treatment recommendations for patients. Large language models (LLMs) can catalyze MTB recommendations, decrease human error, improve accessibility to care, and enhance the efficiency of precision oncology.

**Objective:**

In this study, we aimed to investigate the efficacy of LLM-generated treatments for MTB patients. We specifically investigate the LLMs’ ability to generate evidence-based treatment recommendations using PubMed references.

**Methods:**

We built a retrieval augmented generation pipeline using PubMed data. We prompted the resulting LLM to generate treatment recommendations with PubMed references using a test set of patients from an MTB conference at a large comprehensive cancer center at a tertiary care institution. Members of the MTB manually assessed the relevancy and correctness of the generated responses.

**Results:**

A total of 75% of the referenced articles were properly cited from PubMed, while 17% of the referenced articles were hallucinations, and the remaining were not properly cited from PubMed. Clinician-generated LLM queries achieved higher accuracy through clinician evaluation than automated queries, with clinicians labeling 25% of LLM responses as equal to their recommendations and 37.5% as alternative plausible treatments.

**Conclusions:**

This study demonstrates how retrieval augmented generation–enhanced LLMs can be a powerful tool in accelerating MTB conferences, as LLMs are sometimes capable of achieving clinician-equal treatment recommendations. However, further investigation is required to achieve stable results with zero hallucinations. LLMs signify a scalable solution to the time-intensive process of MTB investigations. However, LLM performance demonstrates that they must be used with heavy clinician supervision, and cannot yet fully automate the MTB pipeline.

## Introduction

### Background

Precision oncology is a rapidly evolving field in which patient treatment is determined by the DNA signature of the tumor. Advancements in genome-sequencing techniques, molecular analysis, and a growing body of clinical trials, have all allowed for the field to integrate more easily into oncology research [1]. Using these techniques to optimize treatment has been shown to be associated with favored clinical outcomes [1]. As a mechanism to facilitate precision oncology, the molecular tumor board (MTB) serves as an approach in which a panel of expert health care providers use their collective knowledge to interpret the genomic profile of an oncology patient and jointly develop an optimal treatment plan given available therapies. Access to MTBs is likely to correlate to improved patient care [1]. The workflow of an MTB is usually as follows: traditionally, before the conference meeting, members of the panel review a list of patients and their corresponding history and characteristics.

**Figure 1:**
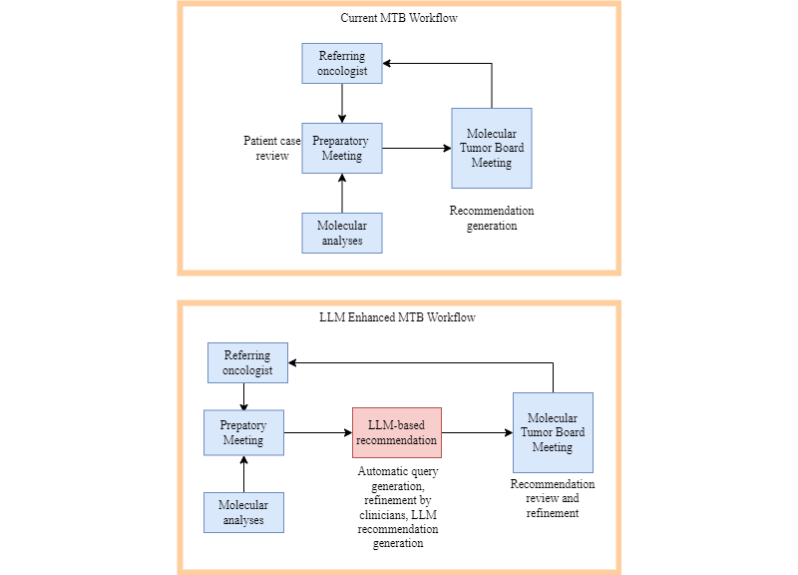
On the top is the current MTB workflow. On the bottom is the LLM-enhanced MTB workflow, in which patient characteristics from the preparatory meeting are used to automatically generate queries. These queries are refined by clinicians and then fed into the LLM for recommendation generation. This recommendation is then reviewed and refined by the MTB panel. LLM: large language model; MTB: molecular tumor board.

During the meeting, members propose targeted therapies and generate a consensus recommendation, which is then received by the treating physician [1]. We propose using large language models (LLMs) to enhance this workflow. The current and proposed LLM-enhanced MTB workflows are shown in Figure 1.

One standing challenge to MTBs is the amount of required resources. Currently, the interpretation of a patient’s molecular profile is a manual process, consisting of searching through various databases, such as OncoKB by Memorial Sloan Kettering Cancer Cancer [2], COSMIC by Wellcome Sanger Institute [3], and PubMed [4]. The process of generating the optimal treatment plan is laborious, and scales poorly, potentially limiting the number of patients that can be reviewed in a given conference. The median MTB experience time, which is the time needed to conduct molecular analysis and hold discussion, was reported in a global analysis published in 2020 to be 38.4 days, with a large range from 12.4 to 86 days [5].

LLMs are models trained on large amounts of textual data that are capable of generating language similar to that of humans. LLMs’ capabilities span a diverse array of tasks, including question-answering, summarization, translation, and conversing. The development and integration of LLMs is advancing rapidly across different sectors. In particular, LLMs demonstrate impressive performance in automated analyses and syntheses of data [6]. As the written text is used heavily in the medical domain for communication between health care providers, between health care providers and patients, and reports on diagnoses, procedures, and patient results, it is an opportune moment for LLM application [7-10]. LLMs have a high potential to improve patient care and the scalability of patient care by increasing medical reasoning, medical knowledge competency, and access to scientific knowledge [11].

The use of MTBs, as stated above, is aided by the advancements in several adjacent fields within oncology, yet its connection to artificial intelligence remains understudied. LLMs have the potential to drastically increase the scalability of MTBs, ease access to information, and prevent human error [12]. LLMs, when used in the proper context with proper understanding, can be a catalyst for precision oncology integration.

### Previous Works

Previous works have demonstrated the feasibility of using natural language processing (NLP) in the field of precision medicine for a plethora of applications, from treatment plans to predictive diagnoses and prognoses. Within precision oncology, researchers have already conducted a study evaluating LLMs for the role they can play in clinical decision-making in the MTB space, although this evaluation does not include experimentation on using different information retrieval (IR), or retrieval augmented generation (RAG) techniques in conjunction with a pretrained LLM [13]. As a result, this study found that LLMs are not suitable to be used in automating the MTB process [14].

Outside the context of MTBs, there has been a substantial amount of research on creating LLMs for the clinical domain. This can be done through different approaches such as generative data augmentation [15], pretraining on medical domain literature [16,17], and fine-tuning on clinical domain data [17-20]. Additionally, these investigations explore different LLMs such as LLaMA, which is open-source, as opposed to GPT, which is proprietary. The result of this difference is that LLaMA can be run locally and therefore offers inherent data security advantages.

Additionally, there are several related works in the field of IR. Such studies investigate how to use NLP and named entity recognition (NER) to analyze medical databases. Through NER, literature can be obtained for a given keyword search, such as a gene, by filtering articles by term presence [21]. One study published the RetriLite framework, in which IR combines with NLP and LLMs in powerful ways to create a functional pipeline in which NLP is used for context analysis and NER, and IR is used for query expansion and relevancy-based ranking [22,23]. RetriLite is an IR framework that capitalizes on NLP to identify relevant articles from domain-specific knowledge bases and extract these sources [22]. This approach focuses on retrieving the highest quality of results, but does not explore the presentation of that output via LLM-produced textual responses.

To summarize, previous works have focused on IR in the medical domain and fine-tuning LLMs for medical domain-specific tasks. Some models that have focused specifically on the interaction and integration of IR and NLP retrieve high-relevancy results. However, the use of LLMs within precision oncology to produce meaningful, evidence-based recommendations remains undetermined. Applying LLMs to MTBs has clear promise in terms of saving time and money, improving accessibility, and increasing the feasibility and scale of MTBs.

### Objectives

This article (1) presents a framework for using LLMs with medical domain knowledge to generate treatment plans for sample cases from MTB conferences, (2) explores the effects of different prompt and query techniques on the LLM, and (3) conducts an evaluation against ground truth historical cases from an MTB annotated by members of the MTB panel.

## Methods

### Ethical Considerations

This study was exempted by the University of Tübingen’s ethics review board due to the exclusive use of nonidentifiable data (Article 2(1) GDPR).

### Constructing the LLM Framework

**Figure 2:**
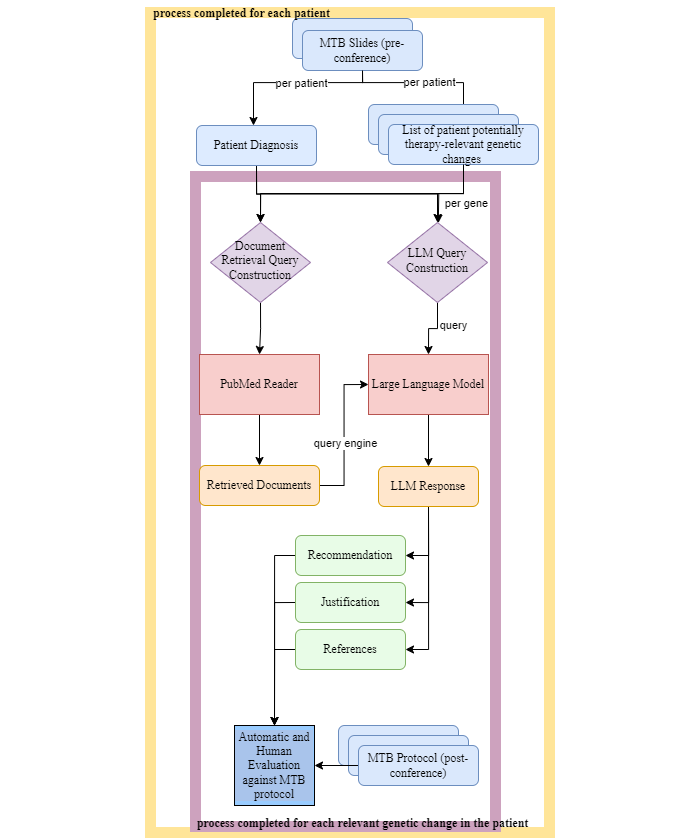
Given the input documents to an MTB conference, we parse the data into actionable queries for each pair of cancer type and genetic mutation. For each patient, we identify the diagnosis and the relevant genetic mutations by filtering the genes that are oncoKB level 1, 2, or 3, as scraped and listed in Table S4, Multimedia Appendix 1. We construct a query for document retrieval and a query for LLM inference. The LLM uses RAG, incorporating documents retrieved by querying the PubMed Data Loader using LlamaIndex, as detailed in the Methods. For each LLM response, we isolate the treatment recommendation, justification, and references and evaluate them against the MTB protocol, which we consider the ground truth. LLM: large language model; MTB: molecular tumor board; RAG: retrieval augmented generation.

To construct the framework that would be used to obtain treatment recommendations for sample MTB cases, we first selected a baseline model to use. This decision was driven by experimenting with various open-source LLMs and selecting the LLM that had the best performance on treatment recommendations without any downstream fine-tuning. Prompt experimentation on different LLMs is shown in Table S1 in Multimedia Appendix 1. The selected model was Llama-2-7b-chat [24]. In addition to its baseline performance prior to fine-tuning, as mentioned in Previous Works, LLaMA also offers inherent security advantages over other non-open-source models such as GPT-4.0. We also experimented with various hyperparameters for this model. The optimal hyperparameters are shown in Table S2 in Multimedia Appendix 1. A full workflow is shown in Figure 2.

To interface with the LLM as a search engine and to integrate PubMed data, we used LlamaIndex [25]. LlamaIndex is an orchestration framework for incorporating custom data into an LLM. It injects data from custom sources into the LLM, embeds the data as vectors, and allows the data to be queried by storing it in a vectorized database. Using LlamaIndex allows us to use RAG to harness the capabilities of an LLM for domain-specific tasks. We then feed the PubMed papers from LlamaIndex into the LLM to be used for RAG [25]. This is highlighted in red and orange in Figure 2. A full workflow of the LLamaIndex orchestration, detailing data selection and literature retrieval is shown in Figure S1 in Multimedia Appendix 1.

To determine the ideal query for the PubMed-adjusted LLM, we experimented with different query types both for document retrieval and for text generation. We determined the optimal document retrieval query from the PubMed data loader would be the name of the broader cancer type, and the genetic mutation to be investigated. This is shown in Table 1, and further discussed in the discussion. Experimentation of various queries with the same LLM pipeline is shown in Table S3 in Multimedia Appendix 1. The genetic mutations to be further queried were determined as follows: for a given patient’s list of genetic mutations that are included in the briefing document sent out to MTB panelists before the meeting, any genetic mutation from that list that is flagged on OncoKB as “level of evidence one, two, or three” (under their original name or alternate name) is further queried. These 3 levels distinguish varying degrees of available clinical evidence. Level 1 drugs are Food and Drug Administration (FDA) recognized biomarkers that are predictive of response to an FDA-approved drug in a specific indication. Level 2 drugs are standard care biomarkers recommended by the National Comprehensive Cancer Network (NCCN) or other professional guidelines predictive of response to an FDA-approved drug. Level 3 drugs have compelling clinical evidence supporting the biomarker as being predictive of response to a drug. Other drugs exist on OncoKB with evidence categorized as biological rather than clinical, with hypothetical therapeutic implications based on preliminary data, or without a determined level of evidence [2]. The resulting full list of genes is shown in Table S4 in Multimedia Appendix 1. For example, “Occult metastatic melanoma NRAS” would be a query for document retrieval. NRAS is listed on OncoKB as a level 1 gene. We define “broader cancer type” as the cancer diagnosis excluding the stage of the cancer, the location of the tumor, or other additional information that may have been included in the diagnosis. This is because when the query exceeds a certain length, the results are too noisy for meaningful articles to be retrieved. Then, a Vector Store Index is constructed from the retrieved documents, which then serves as a query engine. The Vector Store Index stores each document node and corresponding embedding in a Vector Store, allowing a query to fetch the most similar nodes. The optimal query for the LLM uses the full name of the cancer, provided by the MTB. The query is shown in Table 1.

**Table 1:** Input illustration for document retrieval and large language model (LLM) querying. For the Cancer Type Short, we input the type of cancer without further specifications such as stage or tumor location. For Cancer Type Long, we input the exact diagnosis listed in the molecular tumor board (MTB). The gene mutation is inputted as it is listed on OncoKB.

Task	Query
Document retrieval	Cancer Type Short _+ “ ” +_ Gene Mutation
LLM response	*A patient has*_+_ Cancer Type Long _+_ *, and the*_+_ Gene Mutation _+_ *gene mutation. What is the* *best therapy treatment for this patient? Cite the PubMed sources you used to inform your response.*

The results from these queries are in the form of text paragraphs. Each paragraph states the recommendation, provides justification, and cites sources. Including justification in the recommendation allows for chain-of-thought reasoning, which has been shown to improve LLM performance [26]. The number of sources to cite is unspecified in the response. Asking the LLM to cite sources has many shown benefits including allowing the reader to fact-check the LLM response and improving faithfulness and decreasing hallucinations by forcing LLMs to justify their recommendations [27].

### Evaluation Set Construction From Historical MTB Cases

To evaluate LLM recommendations, we created a dataset by retrieving previous cases presented at MTB conferences at the Center for Personalized Medicine at the Universitätsklinikum Tübingen [4]. This was done by manually evaluating case reports given to the panelists before the conference to extract lists of genetic mutations and the cancer diagnosis for each patient to create the queries. As this is not an end-to-end automated product, but a feasibility study of LLM capacities, we decided to manually extract genetic mutations and diagnoses to ensure the accuracy of queries. We further acknowledge that the integration of the case reports into the LLM directly is an important undertaking for future implementations. Then, results were collected from the LLM and compared against the ground-truth responses produced at the conference. The evaluation was conducted by a member of the MTB.

## Results

For each cancer type + genetic mutation pair, we generated 2 different treatment recommendations using the LLM. As shown in Table 1, we do not instruct the LLM to cite a specified number of sources. As a result, the number of sources referenced in one LLM response varied drastically across queries. The number of articles retrieved ranged from 1 to 12 across the 40 total LLM responses (2 unique retrievals per query).

A full histogram of the number of retrieved articles is shown in Figure S2 in Multimedia Appendix 1. Each citation in an LLM response was categorized into one of four types.

1. Hallucination: No article with that title or citation is findable on the internet.
2. Non-PubMed: The article is findable on the internet, but not on PubMed.
3. Inferred-PubMed: The title differed slightly from a title that does exist on PubMed, and the author and journal were identical to what was indicated in the response. Examples of citations labeled as Inferred-PubMed are shown in Table 2.
4. PubMed: The exact article is findable on PubMed.

**Table 2:** Examples of citations labeled as Inferred-PubMed.

LLM Citation	Ground truth citation
Mauduit D, et al (2021). Long and short enhancers in melanoma cell states are enriched in cell-type–specific enhancer function. Elife, 10, e71735.	Mauduit D, Taskiran II, Minnoye L, de Waegeneer M, Christiaens V, Hulselmans G, Demeulemeester J, Wouters J, Aerts S. Analysis of long and short enhancers in melanoma cell states. Elife. 2021 Dec 7;10:e71735. doi: 10.7554/eLife.71735. PMID: 34874265; PMCID: PMC8691835
The study by Maesaki et al (1999) published in the journal Mol Cell found that the structural basis of Rho effector recognition revealed by the crystal structure of human RhoA complexed with the effector domain of PKN/PRK1, which provides insight into the mechanism of resistance to anti-HER2 therapies.	Maesaki R, Ihara K, Shimizu T, Kuroda S, Kaibuchi K, Hakoshima T. The structural basis of Rho effector recognition revealed by the crystal structure of human RhoA complexed with the effector domain of PKN/PRK1. Mol Cell. 1999 Nov;4(5):793-803. doi:10.1016/s1097-2765(00)80389-5. PMID: 10619026.

The complete categorization of articles is shown in Figure S3 in Multimedia Appendix 1. The overall categorization of articles in shown in Table 3. The categorization of articles was conducted through manual human evaluation of retrieved articles. The majority, 76% of retrieved articles, were findable on PubMed, while 17% of articles were hallucinations. Accounting for the final quantity of retrieved articles, 3% of articles were non-PubMed and 3% were PubMed-Inferred. This demonstrates the majority of citations were credible. The cited articles from beyond PubMed demonstrate that the LLM may be using prelearned knowledge, instead of entirely retrieval augmented knowledge, to generate responses, despite prompt instructions.

**Table 3:** The breakdown of all large language model (LLM) retrieved references into types: hallucination, non-PubMed, Inferred-PubMed, and PubMed.

Reference classification	Retrieved LLM references, %
PubMed	76.0
Hallucination	17.1
Inferred-PubMed	3.4
Non-PubMed	3.4

For each LLM response, we evaluated whether at least one of the referenced articles contained the cancer type mentioned in the query. If this was the case, we assigned a binary point to this LLM response. We performed the same search with regard to gene mutation, assigning a binary point if the gene mutation was present in at least one of the referenced articles. We also evaluated whether at least one of the referenced articles had presented the ground truth protocol that the reference MTB had recommended. Similarly, this was awarded a binary point. Last, we evaluated whether the LLM response itself mentioned the Keyword treatment recommendation (eg, “PARP inhibitor” or “olaparib”), also worth one binary point. Therefore, every LLM response’s accuracy was ranked on a scale of 0 to 4, where 0 meets none of the criteria above, and 4 meets all of the criteria above. As previously stated, 2 unique LLM responses were recorded per query. This was motivated by the stochastic nature of LLMs, to demonstrate how consecutive identical queries can yield different outcomes. For each response, we evaluated the above 4 characteristics of the response. The results of this accuracy calculation are shown in Table 4. The accuracy calculation on a 4-point scale for each individual LLM response is shown in Figure S3 in Multimedia Appendix 1. The increasing accuracy (from keyword being present to cancer type being mentioned) is expected in Table 4, as the evaluation metric increasingly broadens. Therefore, the lowest accuracy (25%) corresponds to the treatment name being present in the response, whereas the highest accuracy (82.5%) corresponds to the patient’s cancer type being present in at least one referenced article of the response.

**Table 4:** The overall accuracy of large language model (LLM) responses compared with ground-truth molecular tumor board recommendations.

Patient characteristic	LLM responses that include patient characteristic, %
Keyword in response	25
Protocol in at least one referenced article	45
Gene mutation in at least one referenced article	50
Cancer type in at least one referenced article	82.5

The LLM responses and retrieved articles were presented to an MTB. MTB members were asked to annotate the responses and retrieved articles according to the following criteria. For each article (excluding hallucinations), we asked the MTB members to label it as Not Relevant or Relevant. Not relevant articles should have nothing to do with the case being presented. For each LLM response, we asked MTB members to label the text response as either Off-base, Alternative, or Reference. We define the three LLM response labels as follows:

1. Off-base: This treatment suggestion does not make sense as a possible treatment for the patient.
2. Reference: This treatment is what was suggested at the MTB conference.
3. Alternative: This is not the treatment suggested in the MTB conference, but it is a potential alternative treatment route for the patient.

After collecting feedback from one clinician, we asked them to provide the query with which they themselves would have prompted the LLM. We obtained a second round of LLM responses and collected corresponding feedback on these responses. The results from this evaluation are shown in Table 5. The LLM prompted with automatically generated queries retrieved treatment recommendations equal to that of clinicians in 32% of cases, and alternative plausible treatment recommendations in 16% of cases. When the LLM was prompted (for the same cases) with queries crafted by clinicians, the recommendations were equal to that of clinicians in 25% of cases, and alternative plausible treatment recommendations in 38% of cases. Clinician-generated queries yielded more accurate LLM responses and more relevant publication citations.

**Table 5:** Annotation from molecular tumor board (MTB) members of large language model (LLM) responses and retrieved articles. Automated queries were constructed by combining a gene name and cancer diagnosis. Clinician queries were created by an MTB member for the same case.

Classification		Percentage from automated queries, %	Percentage from clinician queries, %
**Retrieved article feedback**			
	Not relevant	87.02	67.78
	Relevant	12.98	32.22
**LLM response feedback**			
	Off-base	52.63	37.50
	Alternative	15.79	37.50
	Reference	31.58	25.00

Additionally, feedback from clinicians regarding some LLM responses indicates that there is more nuance to MTB treatment recommendations than solely what was included in the LLM query. For example, in the case of a patient with adenocarcinoma of the esophagogastric junction and the ERBB2 amplification, clinician feedback noted that the MTB recommendation (Trastuzumab-Deruxtecan) was based on the pathology HER2 DAKO 3+ Score, which was due to the ERBB2 Amplification. Furthermore, the recommendation was made because the patient had already previously received Trastuzumab. This information was only derived from interaction with the clinical team and was not available to the LLM. Consequently, our experiments should be considered a lower bound of how well RAG-enhanced LLMs could work in the MTB space when fully integrated into care systems such as electronic health records. We opted for these evaluation metrics as they offer a more informative approach than fully automated metrics such as ROUGE scores, which track topicality at a high level of abstraction. The evaluation methods we have chosen use explicit human judgments and are possible given the small size of our dataset.

## Discussion

### Summary

We developed a RAG-enhanced LLM to streamline the treatment recommendation process for MTB conferences. This provided a pipeline to generate treatment recommendations for MTB patients quickly while maintaining a rigorous evidence retrieval stage from medical literature. When the LLM is prompted with automatically generated queries to retrieve treatment recommendations, these recommendations were equal to that of clinicians or alternative plausible treatment recommendations in 48% of cases. However, when the LLM was prompted with clinician-generated queries, recommendations were equal to that of clinicians or alternative plausible treatment recommendations in 63% of cases. Importantly, this demonstrates that high-quality treatment recommendations can in some cases be achieved by the LLM without the manual and time-consuming tasks of conducting human literature reviews and synthesizing retrieved documents. This is even more promising when clinicians play a role in query generation [12]. However, this is not always the case, and some retrieved articles used as evidence are highly irrelevant, demonstrating a disconnect between recommendations and citations. Additionally, hallucinations remain a persistent problem in LLM responses, accounting for 17.1% of cited sources. LLMs have the potential to make the MTB workflow more efficient, and scalable, which can lead to greater access to personalized patient care. To our knowledge, this is the first attempt to apply RAG to enhance LLMs for MTB use.

### Key Findings

This study demonstrates several key findings. First, LLMs can be powerful tools to aid in clinician decision-making on MTBs. However, the use of NLP-assisted MTB recommendations necessitates oversight by MTB members at multiple stages of the pipeline, including query generation as well as recommendation assessment prior to presentation to a patient. Second, the references retrieved for a given LLM response do not align directly with the textual response generated by the LLM. This suggests that rather than all of the responses being produced through RAG approaches, LLM memory also plays a significant role in its decision-making. This role must be further explored and understood so as to mitigate unintended consequences of using pretrained LLMs to generate patient treatment recommendations. Last, it is essential to consider the drawbacks of LLMs (as they currently function) in the clinician domain: namely, the potential dissemination of misinformation through hallucinatory responses that must be properly vetted before being legitimized in patient treatment. Strategies to mitigate hallucinations are imperative to ensure the trustworthiness of LLM-generated treatment suggestions.

It is important to note that the results from our study are in slight disagreement with that of the study conducted using pretrained LLMs on fictional patient vignettes [14]. Their study noted how the accuracy of provided LLM references was often a reason why the LLM itself was deemed unfit for the cause. We believe our study poses a potential solution to this, through the addition of RAG. Therefore, this new approach can advance the field by demonstrating the potential for a more robust LLM-enhanced MTB pipeline that decreases hallucination rates. Hallucinations still pose a serious problem in LLM implementation in health care settings. In this feasibility study, our goal is to assess the extent of this issue and benchmark existing components, rather than resolve it.

### Limitations

Limitations of this study include reproducibility of LLM responses, scope of query inputs, evaluation technique, and generalizability.

#### Reproducibility

Given the dynamic nature of LLMs, it is not guaranteed to achieve consistent responses, even when the same prompt is applied [11]. Additionally, we observed different LLMs can yield different results, given the same prompt. Results from this are shown in Table S1 in Multimedia Appendix 1. We observed changes in LLM recommendations when we reran inference numerous times with identical prompts on the same model. Due to this, it is crucial to document all model parameters, prompt templates, and model versions when experimenting with LLMs in the medical domain. The goal of this study is to observe and quantify the extent of such behavior in LLMs. We leave the challenging task of addressing these issues for future work.

#### Scope of Queries

Currently, when we extract information from the patient’s genetic characteristics provided prior to the conference, we are identifying and querying genetic mutations that are listed on OncoKB as level 1, 2, or 3, as shown in Table S4 in Multimedia Appendix 1. This excludes not-categorized genes as well as genes listed with levels above 3, which may also be listed on the patient’s genetic information. Additionally, a number of treatment recommendations from MTB conferences were motivated by other, non–genetic-mutation characteristics of the patient, such as tumor mutation burden range, homologous recombination deficiency assessment, and human lymphocyte antigen expression levels. These values, while promising biomarkers for responsiveness to certain treatments [28-30] were not available to our models. In future modifications to this algorithm, we hope to extract all patient information pertaining to their genetic mutations and biomarkers so that we can increase the scope and therefore, the robustness, of generated recommendations.

#### Evaluation Technique

Feedback from clinicians suggested that a further evaluation category should be included to account for LLM responses that list a combination treatment of multiple drugs, but the MTB recommended only one of them, or vice versa. Adding this category would improve the robustness of the evaluation.

#### Generalizability

This study is focused on a single comprehensive cancer center. In the future, this approach can be expanded and evaluated at additional sites and in different health care settings to gauge the generalizability and scalability of the proposed methods.

### Future Directions

There are many future directions motivated by this study.

#### Using Local Hospital Data for RAG

First, our model is built on PubMed sources to augment and improve the credibility and accuracy of LLM responses, decrease LLM hallucination rate, and isolate the latest research to inform recommendations. However, by enhancing this system by using local hospital data (ie, using existing local hospital patient history and MTB conference recommendations), we can tune the model to be more accurate within the specific context of the hospital’s resources and history of care. Existing research demonstrates that in-house models can better accommodate the needs of specific centers, therefore enhancing precision medicine across the field [12]. This would also allow for a framework to generalize this approach across various health care settings. Upon expansion to other health care settings, further experimentation would be needed to investigate the scalability of the proposed approach.

#### Increasing Response Relevance and Decreasing Hallucinations Through Isolating IR from LLM Generation, and LLM Architecture Experimentation

A second direction for future research is to use IR approaches as an independent and isolated step prior to LLM interfacing. This approach can retrieve the optimal articles and directly feed those articles into the LLM to perform RAG. Doing so would ensure that the referenced articles are relevant before we even interface with the LLM. This would also likely increase LLM recommendation performance, since the LLM would be basing its recommendation on a small sample of preselected relevant articles. Through this approach, we could include additional evidence such as scientific meeting abstracts and posters in the searched literature as they provide the most up-to-date trial information, which was a point of feedback made by clinicians. This is motivated by the observation that LLM memory plays a significant role in decision-making, potentially leading to unintended consequences. The misalignment between the sources and response text in some cases can be mitigated in future studies by more robust filtering and contextualization of search results. In order to further understand the role of memory in LLM decision-making, work is being done in the field of mechanistic interpretability to create human-readable explanations of artificial intelligence–generated representations [31]*.* Another potential experiment to reduce hallucinations and overall increase the accuracy of responses involves using ensemble learning, rather than a single LLM, to harness the power of multiple models. Not only does this have the potential to improve performance, but it also could improve the reliability of results [32].

#### Experiment Further With Prompting Strategies

Another interesting area of future research lies in diverse prompting strategies. In our experimentation, by adjusting the query slightly, different results are generated by the LLM. Different directions include asking the LLM to rank different treatments, list multiple treatments, or decide between 2 given treatments. Different prompting strategies have been shown to augment LLM text generation, and optimizing prompting strategies can enhance the accuracy, comprehensibility, and quality of LLM responses [33,34]. We can also make the query broader or more specific, such as asking for a general treatment, versus asking for a specific drug name for a targeted therapy approach. The genetic mutation can also be further specified to include details regarding therapy-relevant copy number variations (amplifications and deletions), fusion genes, and rearrangements. Additionally, our clinician feedback suggested a further experiment of feeding a clinician-generated treatment recommendation into the LLM and directly asking the LLM whether, under consideration of this MTB recommendation, further treatment recommendations are suggested by the LLM. Clinicians also noted that, while MTB decisions ideally are based on reports from the same tumor type (m1 evidence), they also recommend treatments successful in other tumor entities with the same genetic alteration (m2 evidence). Therefore, we could expand the query-process into a 2-step approach, in which after looking for m1 evidence, we query for results regarding any cancer type. As clinician feedback is a crucial part of the proposed pipeline, we would like to further experiment with diverse prompting strategies to optimize LLM text generation that directly uses this feedback. In addition to broader human feedback, using strategies such as chain-of-thought prompting and prompt tuning could further improve the accuracy of generated results [35,36].

#### Improve the Interpretability and Transparency of RAG-Enhanced LLMs to Facilitate Trust Among Clinicians and Patients

There are several existing methods that can be used as tools in LLM evaluation, such as gradient tracing, Shapley values, probing, or casual mediation. For example, Shapley values demystify LLM decision-making by quantitatively determining how much each word in the input text contributes to the LLM output text. This can be used to detect bias by identifying features that disproportionately affect model outputs [37]. This could be used, along with the above-mentioned tools, to increase transparency of LLM decision-making within the MTB treatment recommendation context. Additionally, to improve the LLM’s ability to understand the nuances of the MTB treatment recommendations, and therefore create more context-driven responses, we can use existing methods such as reinforcement learning from human feedback, targeted instruction tuning, and grounding LLMs [3,38,39].

#### Analyze Change in Efficiency of MTB Pipeline

Last, with this study, we demonstrated the feasibility of incorporating LLMs into the MTB pipeline. However, it remains to be shown that this translates to increased efficiency. In a future study, we hope to analyze the effort needed to manually operate the MTB pipeline, and contrast that with that of the LLM-enhanced pipeline. This study evaluates existing open-source components of LLMs in the MTB context, rather than building production-ready systems. Therefore, this study measures the feasibility of LLMs as they currently exist in the MTB pipeline. Our recommendation based on this study is to continue working toward fixing the observed issues before moving on to prospective evaluation and clinical integration in real-world settings.

### Conclusions

In this study, we developed a RAG approach to LLM text generation to develop treatment recommendations for MTB patients. Specifically, we evaluated the articles referenced in the LLM response, as well as the treatment recommendation itself. This study demonstrates that LLMs have the potential to be harnessed for rapid treatment plan generation based on patient DNA signature and diagnosis, generating responses comparable to that of clinicians and highlighting the potential of LLMs in precision oncology. The use of such an approach has a high feasibility at scale, as opposed to current manual MTB investigations. However, LLMs are not currently suitable to fully automate the MTB process and must be used with supervision from clinicians at each stage of their deployment. We have shown the feasibility of the implementation of LLMs in the MTB pipeline, and in future work, we hope to analyze how this feasibility has the potential of further translating into increased efficiency.

## Appendix

Multimedia Appendix 1 Supplementary tables and figures.

## Abbreviations

FDA: Food and Drug Administration
IR: information retrieval
LLM: large language model
MTB: molecular tumor board
NER: named entity recognition
NLP: natural language processing
RAG: retrieval augmented generation

## References

[1] Tsimberidou AM, Kahle M, Vo HH, Baysal MA, Johnson A, Meric-Bernstam F (2023). Molecular tumour boards - current and future considerations for precision oncology. Nat Rev Clin Oncol.

[2] Welcome to OncoKB. OncoKB.

[3] COSMIC: catalogue of somatic mutations in cancer. Wellcome Sanger Institute.

[4] PubMed. National Center for Biotechnology Information.

[5] Luchini C, Lawlor RT, Milella M, Scarpa A (2020). Molecular tumor boards in clinical practice. Trends Cancer.

[6] Naveed H, Khan AU, Qiu S, Saqib M, Anwar S, Usman M, Akhtar N, Barnes N, Mian A (2023). A comprehensive overview of large language models. arXiv:2307.06435.

[7] Locke S, Bashall A, Al-Adely S, Moore J, Wilson A, Kitchen GB (2021). Natural language processing in medicine: a review. Trends Anaesth Crit Care.

[8] Ahmed U, Iqbal K, Aoun M, Khan G (2023). Natural language processing for clinical decision support systems: a review of recent advances in healthcare. J Intell Connectivity Emerging Technol.

[9] Aramaki E, Wakamiya S, Yada S, Nakamura Y (2022). Natural language processing: from bedside to everywhere. Yearb Med Inform.

[10] Hossain E, Rana R, Higgins N, Soar J, Barua PD, Pisani AR, Turner K (2023). Natural language processing in electronic health records in relation to healthcare decision-making: a systematic review. Comput Biol Med.

[11] Clusmann J, Kolbinger FR, Muti HS, Carrero ZI, Eckardt J, Laleh NG, Löffler CML, Schwarzkopf S, Unger M, Veldhuizen GP, Wagner SJ, Kather JN (2023). The future landscape of large language models in medicine. Commun Med (Lond).

[12] Tamborero D, Dienstmann R, Rachid MH, Boekel J, Lopez-Fernandez A, Jonsson M, Razzak A, Braña I, De Petris L, Yachnin J, Baird RD, Loriot Y, Massard C, Martin-Romano P, Opdam F, Schlenk RF, Vernieri C, Masucci M, Villalobos X, Chavarria E, Balmaña J, Apolone G, Caldas C, Bergh J, Ernberg I, Fröhling S, Garralda E, Karlsson C, Tabernero J, Voest E, Rodon J, Lehtiö J (2022). The molecular tumor board portal supports clinical decisions and automated reporting for precision oncology. Nat Cancer.

[13] Patrick Lewis P, Perez E, Piktus A, Petroni F, Karpukhin V, Goyal N, Küttler H, Lewis M, Yih WT, Rocktäschel T, Riedel S, Kiela D (2005). Retrieval-augmented generation for knowledge-intensive nlp tasks. arXiv:2005.11401.

[14] Benary M, Wang XD, Schmidt M, Soll D, Hilfenhaus G, Nassir M, Sigler C, Knödler M, Keller U, Beule D, Keilholz U, Leser U, Rieke DT (2023). Leveraging large language models for decision support in personalized oncology. JAMA Netw Open.

[15] Guo Z, Wang P, Wang Y, Shangdi Yu S (2023). Improving small language models on pubmedqa via generative data augmentation. arXiv:2305.07804.

[16] Luo R, Sun L, Xia Y, Qin T, Zhang S, Poon H, Liu T (2022). BioGPT: generative pre-trained transformer for biomedical text generation and mining. Brief Bioinform.

[17] Wu C, Lin W, Zhang X, Zhang Y, Xie W, Wang Y (2024). PMC-LLaMA: toward building open-source language models for medicine. J Am Med Inform Assoc.

[18] Gema AP, Minervini P, Daines L, Hope T, Alex B (2023). Parameter-efficient fine-tuning of llama for the clinical domain. arXiv:2307.03042.

[19] Karkera N, Acharya S, Palaniappan SK (2023). Leveraging pre-trained language models for mining microbiome-disease relationships. BMC Bioinf.

[20] Rasmy L, Xiang Y, Xie Z, Tao C, Zhi D (2021). Med-BERT: pretrained contextualized embeddings on large-scale structured electronic health records for disease prediction. NPJ Digit Med.

[21] Chen HO, Lin PC, Liu CR, Wang CS, Chiang JH (2021). Contextualizing genes by using text-mined co-occurrence features for cancer gene panel discovery. Front Genet.

[22] Zeng J, Cruz-Pico CX, Saridogan T, Shufean MA, Kahle M, Yang D, Shaw K, Meric-Bernstam F (2022). Natural language processing-assisted literature retrieval and analysis for combination therapy in cancer. JCO Clin Cancer Inform.

[23] Hamamoto R, Koyama T, Kouno N, Yasuda T, Yui S, Sudo K, Hirata M, Sunami K, Kubo T, Takasawa K, Takahashi S, Machino H, Kobayashi K, Asada K, Komatsu M, Kaneko S, Yatabe Y, Yamamoto N (2022). Introducing AI to the molecular tumor board: one direction toward the establishment of precision medicine using large-scale cancer clinical and biological information. Exp Hematol Oncol.

[24] Touvron H, Martin L, Stone K, Albert P, Almahairi A, Babaei Y, Bashlykov N, Batra S, Bhargava P, Bhosale S, Bikel D, Blecher L, Ferrer CC, Chen M, Cucurull G, Esiobu D, Fernandes J, Fu J, Fu W, Fuller B, Gao C, Goswami V, Goyal N, Hartshorn A, Hosseini S (2023). Llama 2: open foundation and fine-tuned chat models. arXiv:2307.09288.

[25] Build AI knowledge assistants over your enterprise data. LlamaIndex.

[26] Wei J, Wang X, Schuurmans D, Bosma M, Ichter B, Xia F, Chi E, Le Q, Zhou D (2023). Chain of thought prompting elicits reasoning in large language models. arXiv:2201.11903.

[27] Gao T, Yen H, Yu J, Chen D (2023). Enabling large language models to generate text with citations. arXiv:2305.14627.

[28] Ma X, Zhang Y, Wang S, Yu J (2021). Predictive value of tumor mutation burden (TMB) with targeted next-generation sequencing in immunocheckpoint inhibitors for non-small cell lung cancer (NSCLC). J Cancer.

[29] Jung J, Heo YJ, Park S (2023). High tumor mutational burden predicts favorable response to anti-PD-(L)1 therapy in patients with solid tumor: a real-world pan-tumor analysis. J Immunother Cancer.

[30] Budczies J, Kluck K, Beck S, Ourailidis I, Allgäuer M, Menzel M, Kazdal D, Perkhofer L, Kleger A, Schirmacher P, Seufferlein T, Stenzinger A (2022). Homologous recombination deficiency is inversely correlated with microsatellite instability and identifies immunologically cold tumors in most cancer types. J Pathol Clin Res.

[31] Bereska L, Gavves E (2024). Mechanistic interpretability for AI safety --a review. arXiv:2404.14082.

[32] Yang H, Li M, Zhou H, Xiao Y, Fang Q, Zhang R (2023). One LLM is not enough: harnessing the power of ensemble learning for medical question answering. medRxiv.

[33] Bsharat SM, Myrzakhan A, Shen Z (2023). Principled instructions are all you need for questioning llama-1/2, gpt-3.5/4. arXiv:2312.16171.

[34] Lyu H, Jiang S, Zeng H, Xia Y, Wang Q, Zhang S, Chen R, Leung C, Tang J, Luo J (2023). Llm-rec: personalized recommendation via prompting large language model. arXiv:2307.15780.

[35] Jason Wei J, Xuezhi Wang X, Dale Schuurmans D, Maarten Bosma M, Brian Ichter B, Fei Xia F, Ed Chi E, Quoc Le Q, Denny Zhou D (1903). Chain-of-thought prompting elicits reasoning in large language models. arXiv:2201.11903.

[36] Lester B, Al-Rfou R, Constant N (2021). Constant, the power of scale for parameter-efficient prompt tuning. arXiv:2104.08691.

[37] Brown NB (1943). Enhancing trust in llms: algorithms for comparing and interpreting llms, 2024. arXiv:2406.01943.

[38] Chaudhari S, Aggarwal P, Murahari V, Rajpurohit T, Kalyan A, Narasimhan K, Deshpande A, da Silva BC (2024). RLHF deciphered: a critical analysis of reinforcement learning from human feedback for llms. arXiv:2404.08555.

[39] Xia M, Malladi S, Gururangan S, Arora S, Chen D (2024). Less: Selecting influential data for targeted instruction tuning. arXiv:2402.04333.

