# Supplementary material for: Retrieval Augmented Therapy Suggestion for Molecular Tumor Boards: Algorithmic Development and Validation Study
Source: J Med Internet Res. 2025 Mar 5;27:e64364. doi: 10.2196/64364 (PMC11923455; doi:10.2196/64364)
Supplement: Multimedia Appendix 1 [file jmir_v27i1e64364_app1.docx]

APPENDIX

This is a Multimedia Appendix to a full manuscript published in the J Med Internet Res.

Figure S1. Data selection and literature retrieval were both orchestrated by the LLamaIndex framework. In terms of data selection, we utilized the LlamaIndex PubMedReader. The PubMedReader receives as input a search query, and returns as output a list of Documents of the top corresponding articles on PubMed through use of the BioC API. This becomes the input for the VectorStoreIndex to form an Index, a data structure that allows for quick retrieval of relevant content for a user query, which is in turn used as to form a Query Engine. The Query Engine takes as input a natural language query, and returns a text rich response. All of these data structures are within the LlamaIndex library.


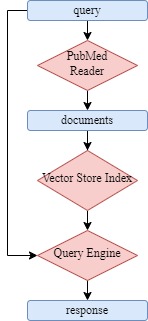


Figure S2. The number of references included in each LLM response for the 40 retrievals performed (two unique retrievals per query).
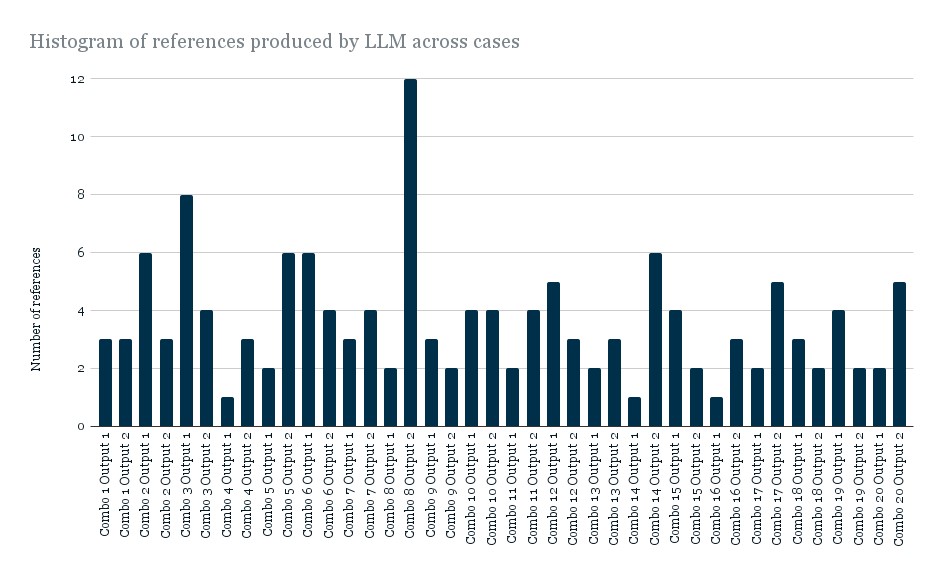


Figure S3. Accuracy of each individual LLM response, ranked on a 4 point scale, where each binary point signifies the presence of a certain patient characteristic in the LLM textual response as well as retrieved references.


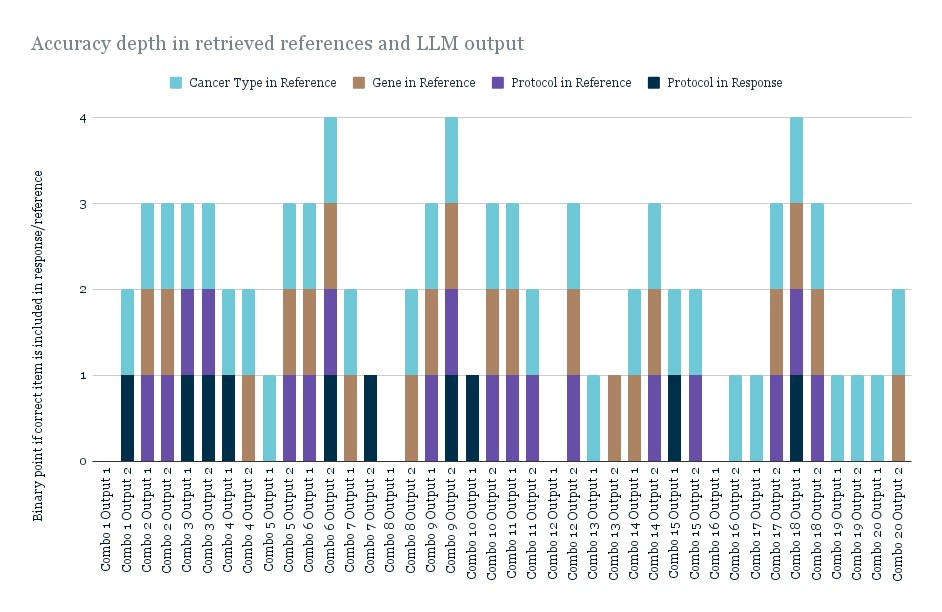


Figure S4. The number of references cited in each LLM response for the 40 retrievals performed. References are categorized by one of four types: 1. Hallucination: No article with that title or citation is findable on the internet. 2. Non-PubMed: the article is findable on the internet, but not on PubMed. 3. Inferred-PubMed: the title differed slightly (by a few words) from a title that does exist on PubMed, and the author and journal in the corresponding PubMed citation were identical to what was indicated in the response. 4. PubMed: the exact article is findable on PubMed.
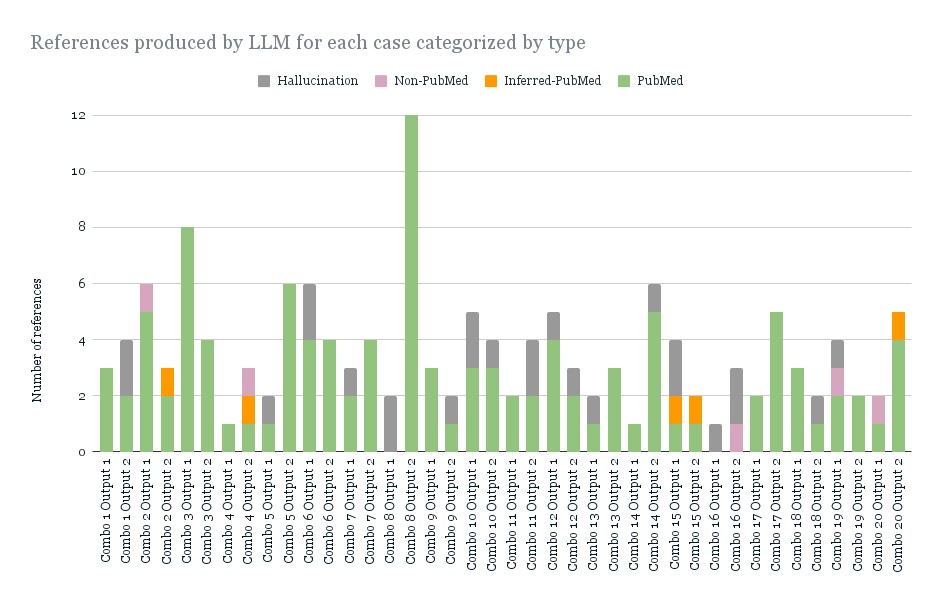


Table S1. The results of prompting different LLMs with the same prompt. After running this prompt with various gene mutations and cancer types through these LLMs, we determined that, prior to any fine tuning, LLaMA best corresponded to the outputs generated by clinicians.

| LLM | Response to prompt: *A patient has adenocarcinoma of the esophagogastric junction and the ERBB2 gene mutation. What is the best therapy treatment for this patient?* |
| --- | --- |
| GPT-3.5  [(https://chat. openai.com/)](https://chat.openai.com/) | The best therapy treatment for a patient with adenocarcinoma of the esophagogastric junction and the ERBB2 gene mutation would typically involve targeted therapy with drugs that specifically inhibit the ERBB2 (also known as HER2) pathway.  One such drug commonly used in this scenario is trastuzumab (Herceptin). Trastuzumab is a monoclonal antibody that targets the HER2 receptor, which is overexpressed in some cancers, including certain types of gastric and gastroesophageal junction cancers. By inhibiting the HER2 pathway, trastuzumab can slow down or halt the growth of cancer cells.  Trastuzumab is often used in combination with chemotherapy to maximize its effectiveness. The specific chemotherapy regimen may vary depending on factors such as the patient’s overall health, stage of cancer, and other individual considerations.  It’s important for the patient to discuss treatment options thoroughly with their oncologist, as they will consider various factors to determine the most appropriate and effective course of action. Additionally, ongoing research and clinical trials may provide further insight into potential treatment options for this specific genetic subtype of cancer. |
| LLaMA2 70-B  [(https://www.](https://www.llama2.ai/)  [llama2.ai/)](https://www.llama2.ai/) | The best therapy treatment for a patient with adenocarcinoma of the esophagogastric junction and the ERBB2 gene mutation would be a combination of chemotherapy and targeted therapy. Chemotherapy, such as a regimen containing fluorouracil, paclitaxel, and cisplatin, would be effective in treating the cancer cells. In addition, targeted therapy with trastuzumab (Herceptin) would be beneficial since the ERBB2 gene mutation is present. Trastuzumab is a monoclonal antibody that targets the HER2 protein and inhibits its activity, which is overexpressed in ERBB2-mutated cancers.  It’s important to note that this patient may also be a candidate for immunotherapy, such as pembrolizumab (Keytruda) or nivolumab (Opdivo), which have been shown to be effective in treating various types of cancer, including gastroesophageal junction cancer. However, the use of immunotherapy in this case would depend on the patient’s overall health status, other medical conditions, and other factors.  It’s important to note that the best treatment plan for this patient would be determined by a multidisciplinary team of oncologists, medical oncologists, radiation oncologists, and other healthcare professionals, and would take into account the patient’s overall health status, other medical conditions, and other factors. |
| Claude  2 ([https:](https://claude.ai/)  [//claude.ai/)](https://claude.ai/) | Based on the details provided, the best therapy treatment for this patient would likely be targeted therapy with trastuzumab (Herceptin). Here is a brief explanation:  Adenocarcinoma of the esophagogastric junction (EGJ) refers to a cancer arising from glandular cells near the area where the esophagus joins the stomach.  The ERBB2 (HER2) gene mutation leads to overexpression of the HER2 protein, which promotes cancer cell growth. Overexpression of HER2 is found in about 20-30% of EGJ adenocarcinomas.  Trastuzumab is a monoclonal antibody that specifically targets the HER2 protein. It has been shown to improve outcomes when added to chemotherapy for patients with HER2-positive metastatic EGJ adenocarcinoma.  Based on major clinical trials, the combination of trastuzumab plus chemotherapy (often cisplatin and fluorouracil or capecitabine) is considered a standard first-line therapy for patients with HER2-positive advanced EGJ cancer.  So in summary, targeted anti-HER2 therapy with trastuzumab would be considered the best and most appropriate initial treatment option for this patient with HER2-positive EGJ adenocarcinoma. Let me know if you need any clarification or have additional questions! |

Table S2. The parameters of the Llama-2-7b-chat model. Given our computation resources, we chose the maximum feasible context_window and max_new_tokens. Temperature was set to 0 to decrease variance and to produce more deterministic and focused output, which is most appropriate for our task.

| Hyperparameter | Value |
| --- | --- |
| context_window | 4096 |
| max_new_tokens | 2048 |
| generate_kwargs | {"temperature": 0.0, "do_sample": False } |
| tokenizer_name | "meta-llama/Llama-2-7b-chat-hf" |
| model_name | "meta-llama/Llama-2-7b-chat-hf" |

Table S3. Experimentation of various prompt templates with the same cancer type and gene mutation into the LLM to observe the differences of outputs depending on prompt wording. Full reference lists have been deleted from the displayed responses below. The optimal query was selected after manual evaluation of the responses to see which yielded recommendations most similar to those of the clinicians, which were considered to be ground-truth.

| *Reference Query*: A patient has Esophageal squamous cell carcinoma, and the BRCA2 gene mutation. gene mutation. What is the best therapy treatment for this patient? Cite the PubMed sources you used to inform your response. | Based on the provided context information, the best therapy treatment for a patient with synchronously lymphogenically metastatic squamous cell carcinoma of the distal esophagus and a BRCA2 gene mutation would be a combination of radiation therapy and a PARP inhibitor. According to the literature, PARP inhibitors have shown promise in the treatment of BRCA-mutated cancers, including ovarian and breast cancer. In a phase I trial of olaparib in combination with high-dose radiotherapy with and without concurrent cisplatin for non-small cell lung cancer, the combination of PARP inhibition and radiation therapy was found to be safe and well-tolerated, with evidence of antitumor activity (De Haan et al., 2021). Similarly, in a phase I and pharmacologic study of olaparib in combination with radiotherapy for non-small cell lung cancer, the addition of PARP inhibition to radiation therapy was found to improve progression-free survival in patients with BRCA1/2 mutations (Karam et al., 2018). In addition, a review article by Teknos and colleagues (2021) discussed the potential benefits of combining PARP inhibitors with radiation therapy in the treatment of BRCA-mutated cancers, including esophageal squamous cell carcinoma. The authors noted that PARP inhibitors can enhance the radiosensitization of cancer cells by activating autophagy signaling, which can lead to increased tumor cell death and improved treatment outcomes. Therefore, based on the available evidence, the best therapy treatment for a patient with synchronously lymphogenically metastatic squamous cell carcinoma of the distal esophagus and a BRCA2 gene mutation would be a combination of radiation therapy and a PARP inhibitor, such as olaparib. |
| --- | --- |
| A patient has Esophageal squamous cell carcinoma, and the BRCA2 gene mutation. How best can this patient be treated? | Based on the provided context information and the PubMed sources listed below, the best treatment for a patient with esophageal squamous cell carcinoma (ESCC) who is BRCA2 positive is likely to involve a combination of surgery and chemotherapy.  According to the ESMO Clinical Practice Guidelines for diagnosis, treatment, and follow-up of ESCC, surgery is the primary treatment for resectable ESCC, with a curative resection rate of approximately 50% for T1-T3N0-1 disease [document reference]. However, for patients with BRCA2 mutations, the guidelines recommend considering neoadjuvant chemotherapy before surgery to improve the chances of a curative resection [document reference].  A systematic review and meta-analysis of 14 randomized controlled trials found that neoadjuvant chemotherapy improved the odds of a curative resection in patients with ESCC, particularly for those with BRCA2 mutations [document reference]. Another study found that BRCA2 mutation status was a significant predictor of response to neoadjuvant chemotherapy in patients with ESCC [document reference].  The choice of chemotherapy regimen for BRCA2-positive ESCC is not well established, but platinum-based doublets are commonly used due to their activity against squamous cell carcinomas [document reference]. A phase III trial found that the addition of cetuximab to a platinum-based doublet improved overall survival in patients with ESCC, but the benefit was only observed in patients with BRCA2 mutations [document reference].  In summary, the best treatment for a patient with BRCA2-positive ESCC is likely to involve a combination of surgery and chemotherapy, with neoadjuvant chemotherapy before surgery being considered for patients with BRCA2 mutations. The choice of chemotherapy regimen should take into account the patient's BRCA2 status and other individual factors. |
| A patient has Esophageal squamous cell carcinoma, and the BRCA2 gene mutation. What is the best treatment for this patient? List the title, document id, and abstract of the PubMed articles that support your response. | Based on the provided context, the best treatment for a patient with esophageal squamous cell carcinoma and a BRCA2 mutation would be a combination of chemotherapy and radiation therapy, with a focus on improving survival outcomes. The median overall survival (OS) for patients treated with conventional chemotherapy is approximately 12 months, and systemic therapy, including perioperative chemotherapy, is a standard of care for resectable gastric cancer in most parts of the Western Hemisphere. However, in East Asian countries, neoadjuvant therapy is recommended only for cancers with extensive lymph node involvement, while postoperative chemotherapy is recommended to control residual tumor cells after curative resection.  Therefore, based on the available evidence from PubMed articles, the best treatment for a patient with esophageal squamous cell carcinoma and a BRCA2 mutation would be a combination of chemotherapy and radiation therapy, with a focus on improving survival outcomes. This approach has been shown to be effective in treating esophageal cancer in patients with BRCA2 mutations, and it is consistent with current NCCN and ESMO clinical practice guidelines.  The following are the title, document ID, and abstract of the PubMed articles that support this refined answer: [documents]  In conclusion, based on the available evidence from PubMed articles, the best treatment for a patient with esophageal squamous cell carcinoma and a BRCA2 mutation would be a combination of chemotherapy and radiation therapy, with a focus on improving survival outcomes. This approach is consistent with current NCCN and ESMO clinical practice guidelines and has been shown to be effective in treating esophageal cancer in patients with BRCA2 mutations. |
| A patient has Esophageal squamous cell carcinoma, and the BRCA2 gene mutation. What are the three best treatments for this patient? Cite the pubmed sources you used to inform your response. | Based on the provided context, the three best treatments for a patient with esophageal squamous cell carcinoma (ESCC) who is BRCA2 are:   1. Neoadjuvant chemotherapy: The use of neoadjuvant chemotherapy in ESCC has been shown to improve the likelihood of achieving a complete pathological response in patients with resectable disease, particularly in those with BRCA2 mutations. A systematic review and meta-analysis of 17 studies found that neoadjuvant chemotherapy resulted in a significant improvement in the rate of complete pathological response compared to surgery alone (odds ratio, 2.37; 95% confidence interval, 1.44-3.90). The review also found that the addition of chemotherapy to surgery improved overall survival (hazard ratio, 0.77; 95% confidence interval, 0.63-0.95). 2. Pembrolizumab: Pembrolizumab, an anti-PD-1 therapy, has been shown to be effective in the treatment of ESCC, particularly in patients with BRCA2 mutations. A phase III randomized controlled trial found that pembrolizumab improved overall survival compared to paclitaxel in patients with advanced ESCC (median overall survival, 10.7 months vs. 8.5 months; hazard ratio, 0.73; 95% confidence interval, 0.57-0.95). The trial also found that pembrolizumab was associated with a higher response rate and longer duration of response compared to paclitaxel. 3. Adjuvant chemotherapy: Adjuvant chemotherapy has been shown to improve overall survival in patients with resectable ESCC, particularly in those with BRCA2 mutations. A systematic review and meta-analysis of 14 studies found that adjuvant chemotherapy resulted in a significant improvement in overall survival compared to surgery alone (hazard ratio, 0.73; 95% confidence interval, 0.62-0.86). The review also found that the addition of chemotherapy to surgery improved disease-free survival (hazard ratio, 0.77; 95% confidence interval, 0.65-0.90).   In conclusion, based on the available evidence, the three best treatments for a patient with ESCC who is BRCA2 are neoadjuvant chemotherapy, pembrolizumab, and adjuvant chemotherapy. These treatments have been shown to improve overall survival and response rates in patients with ESCC, and may be particularly beneficial for patients with BRCA2 mutations. |

Table S4. List of Genes on [oncoKB.org](https://www.oncokb.org/) categorized as Level one, Level two, or Level three. There are 53 Level one genes, 25 Level two genes, and 34 Level three genes. However, as some genes appear in multiple lists, there are 66 unique genes listed across the three groups.

| Gene | Level |
| --- | --- |
| ALB1 | 1 |
| ALK | 1 |
| ATM | 1 |
| ATR | 1 |
| BARD1 | 1 |
| BRAF | 1 |
| BRCA1 | 1 |
| BRCA2 | 1 |
| BRIP1 | 1 |
| CDK12 | 1 |
| CHEK1 | 1 |
| CHEK2 | 1 |
| EGFR | 1 |
| ERBB2 | 1 |
| ESR1 | 1 |
| EZH2 | 1 |
| FANCA | 1 |
| FANCL | 1 |
| FGFR1 | 1 |
| FGFR2 | 1 |
| FGFR3 | 1 |
| FLT3 | 1 |
| IDH1 | 1 |
| IDH2 | 1 |
| KIT | 1 |
| KRAS | 1 |
| MET | 1 |
| MLH1 | 1 |
| MRE11 | 1 |
| NBN | 1 |
| NF1 | 1 |
| NRAS | 1 |
| NTRK1 | 1 |
| NTRK2 | 1 |
| NTRK3 | 1 |
| PALB2 | 1 |
| PDGFB | 1 |
| PDGFRA | 1 |
| PDGFRB | 1 |
| PIK3CA | 1 |
| RAD51B | 1 |
| RAD51C | 1 |
| RAD51D | 1 |
| RAD54L | 1 |
| RARA | 1 |
| RET | 1 |
| ROS1 | 1 |
| SMARCB1 | 1 |
| TSC1 | 1 |
| TSC2 | 1 |
| ABL1 | 2 |
| ALK | 2 |
| ARAF | 2 |
| BRAF | 2 |
| BRCA1 | 2 |
| BRCA2 | 2 |
| EGFR | 2 |
| ERBB2 | 2 |
| ESR1 | 2 |
| EZH2 | 2 |
| IDH1 | 2 |
| JAK2 | 2 |
| KIT | 2 |
| KRAS | 2 |
| MAP2K1 | 2 |
| MAP2K2 | 2 |
| MET | 2 |
| NRAS | 2 |
| PALB2 | 2 |
| PDGFRA | 2 |
| PIK3CA | 2 |
| RAF1 | 2 |
| RET | 2 |
| ROS1 | 2 |
| AKT1 | 3 |
| ARAF | 3 |
| BRAF | 3 |
| BRCA1 | 3 |
| BRCA2 | 3 |
| EGFR | 3 |
| ERBB2 | 3 |
| ERCC2 | 3 |
| ESR1 | 3 |
| FGFR1 | 3 |
| FGFR2 | 3 |
| FGFR3 | 3 |
| FLT3 | 3 |
| HRAS | 3 |
| IDH1 | 3 |
| IDH2 | 3 |
| JAK2 | 3 |
| KMT2A | 3 |
| KRAS | 3 |
| MAP2K1 | 3 |
| MAP2K2 | 3 |
| MET | 3 |
| MTOR | 3 |
| NPM1 | 3 |
| NRAS | 3 |
| NRG1 | 3 |
| NTRK1 | 3 |
| NTRK2 | 3 |
| NTRK3 | 3 |
| PTCH1 | 3 |
| RAF1 | 3 |
| RET | 3 |
| ROS1 | 3 |
| TP53 | 3 |
| TSC2 | 3 |
